# Going beyond Quietness: Determining the Emotionally Restorative Effect of Acoustic Environments in Urban Open Public Spaces

**DOI:** 10.3390/ijerph16071284

**Published:** 2019-04-10

**Authors:** Karmele Herranz-Pascual, Itziar Aspuru, Ioseba Iraurgi, Álvaro Santander, Jose Luis Eguiguren, Igone García

**Affiliations:** 1 TECNALIA Research and Innovation, Parque Tecnológico de Bizkaia, Calle Geldo, Edificio 700, 48160 Derio, Bizkaia, Spain; karmele.herranz@tecnalia.com (K.H.-P.); alvaro.santander@tecnalia.com (Á.S.); jluis.eguiguren@tecnalia.com (J.L.E.); igone.garcia@tecnalia.com (I.G.); 2 DeustoPsych, University of Deusto, Unibertsitate Etorb. 24, 48007 Bilbao, Bizkaia, Spain; ioseba.iraurgi@deusto.es

**Keywords:** quietness, soundscape, psychological restoration, emotions, acoustic environment, urban open public spaces, urban design

## Abstract

The capacity of natural settings to promote psychological restoration has attracted increasing research attention, especially with regards to the visual dimension. However, there is a need to extend these studies to urban settings, such as squares, parks or gardens, due to the global trend towards urbanisation, and to integrate the dimension of sound into landscape. Such was the main aim of this study, in which 53 participants assessed four public spaces in Vitoria-Gasteiz (Spain) as part of the CITI-SENSE Project (137 observations were used for analysis). A smartphone application was used to simultaneously collect objective and subjective data. The results show that at the end of the urban environmental experience, there was a statistically significant reduction in negative emotions and perceived stress, and a slight increase in positive emotions. Emotional restoration was mainly associated with prior emotional states, but also with global environmental comfort and acoustic comfort. The soundscape characteristics that contributed to greater emotional restoration and a reduction in perceived stress were pleasantness, calm, fun and naturalness. Therefore, in agreement with previous research, the findings of the present study indicate that besides contributing to the quietness of the urban environment, the urban soundscape can promote psychological restoration in users of these spaces.

## 1. Introduction

Restorative environments enhance or facilitate psychological restoration, and thus contribute to human health and well-being. The most influential theories on this topic are attention restoration theory (ART) developed by Kaplan and Kaplan [1] and the stress recovery theory (SRT), postulated by Ulrich [2,3,4].

ART states that natural environments can restore the cognitive resources that people use in their daily activities (e.g., work, studies, responsibilities). In ART theory, the restorative potential of environments, known as restorativeness, is defined by four fundamental dimensions: (a) “being away”, which refers to a series of perceived characteristics that allow individuals to distance themselves physically or psychologically from that which requires their directed attention; (b) “extent”, which refers to the environmental qualities that invite exploration beyond what is immediately perceived; (c) “fascination”, the perceived characteristics that attract people’s attention (this refers to involuntary attention, which does not require excessive mental exertion); and (d) “compatibility”, which refers to the perception that the environment is consonant with the goals of the person experiencing it [1].

Meanwhile, Ulrich’s theory postulates that despite its adaptative value, the stress response elicited by some life events drains psychological energy and leads to the emergence of a negative emotional state. Thus, a positive affective response to open natural settings enables the individual to recover from fatigue and its negative emotional outcomes.

In recent decades, the study of psychological restoration has attracted considerable research interest in environmental psychology and beyond, although most studies have focused primarily on natural settings (outside urban areas) such as parks and forests. It has been found that exposure to green or blue natural environments (with vegetation and water, respectively) can provide a more effective restorative experience than exposure to artificial urban areas [1,2,3,4,5,6,7,8,9,10]. However, this restorative experience does not occur solely in natural settings, although it is enhanced by them [6,10,11,12], and not all such environments contribute to restoration [13]. In connection with the latter, research conducted by Ojala et al. [14] is particularly noteworthy. It assesses how differences in orientation towards built vs. natural environments as well as noise sensibility affect psychological and physiological restoration in three different urban places.

Very few recent studies have explored the restorative capacity of urban settings, since these are generally approached from a negative point of view, considering cities as settings which may give rise to psychological ill health and social disruption as a result of social, economic, environmental and spatial factors [6,7,8,9,10,11,12,13,14,15,16,17]. Urban settings are consequently seen as more stressful and less attractive than natural ones, and in some way responsible for negative effects that can only be palliated through contact with nature.

There is thus a need to extend research on restorative environments to urban settings in order to determine whether these may also be considered restorative, as some recent studies would seem to suggest [18]. The results of one recent study revealed that participants’ psychological state improved after spending half an hour in one of two selected urban squares [19,20]. Visitors to both squares showed better cognitive performance, reduced negative affect variables (tension-anxiety, anger-hostility, fatigue and stress) and reported an increase in happiness. Consequently, applying the restorative environment approach to cities may be an effective way of ameliorating urban life and contributing to people’s health and well-being.

The United Nations Department of Economic and Social Affairs has reported that the urban population has increased exponentially—from 751 million in 1950 to 4200 million in 2018—and that this trend will continue [21]: currently, 55% of world’s population lives in cities and this number is expected to rise to 68% by 2050. In this context, research on restorative environments could help to improve the health and well-being of people living in an urban world, where the workload (or lack thereof) is so stressful.

Another important aspect of previous research on restorative environments is that it has focused primarily on the visual dimension of nature, as reflected in the terms used to describe them (e.g., contemplation, scene, views, green elements), as well as the dimensions that define them (e.g., extent, being away, fascination). These terms are difficult to apply to senses other than sight; however, perception is a holistic process that integrates information from various senses, including sight and hearing. While it is an important environmental element, with social and aesthetic attributes, the quality of soundscape is one of the key factors for environmental perception in urban public open spaces [22,23,24].

In addition, there is a prevailing tendency to consider the urban acoustic environment solely from the perspective of noise pollution. As a result, studies in this field have focused on its harmful effects on people. In its recent publication “Environmental Noise Guidelines for the European Region” [25], the World Health Organization (WHO) regional office for Europe reported that sufficient scientific evidence was available to quantify the health effects of noise for cardiovascular disease (CVD), which includes ischaemic heart disease (IHD) and hypertension (HT), sleep disturbance (SD), annoyance (HA), hearing loss and tinnitus (HL/T) and cognitive impairment (CI). Noise also exerts an adverse effect on newborns, metabolic function, quality of life, mental health and well-being.

Recent years have witnessed an increase in studies analysing the acoustic environment from a positive perspective, focusing attention on its beneficial effects on people [26,27]. In the city of Rotterdam, where 16 urban parks were assessed, restorative levels were mainly due to the park size and the average noise level [28]. For its part, in Milan it was confirmed that the perceived environmental quality of five urban parks was dependent on the type of soundscape [29,30].

Studies also started to explore the impact of soundscape on restoration [10]. A survey conducted in the city of Sheffield showed that the soundscape of urban parks played a significant role in their restorative experience [31].

This current trend includes the study of restorative aspects linked to the soundscape and new surveys on quiet areas [32]. In fact, the restoration theory has very rarely been addressed as a reference in soundscape studies, apart from Payne et al. [33,34], who included two adjectives in their soundscape scale, grouped in the “pleasantness” dimension, which refer to the known positive perception of “nature” and to the restorative capacity of the soundscape. In other laboratory research, Zhang, et al. [35] reported how the typical urban soundscapes with natural elements in densely populated Chinese cities had significant effects on individuals’ restorative experiences; natural sounds will boost the restoration of the individual’s attention, whereas traffic and machine sounds will have a negative effect [10].

Other projects have assessed the sound environment from the point of view of acoustic comfort [36,37,38,39], thus falling within the field of research on environmental comfort. A “comfortable place” is understood as one that can create a pleasant environmental experience for the people and the communities that use it, carrying out individual or social activities [40].

The acoustic dimension of environmental comfort can be assessed using the concept of soundscape. This concept has been developed within the framework of several European actions and projects (many of which formed part of the COST-Action on “Soundscape of European Cities and Landscapes”) aimed at collecting people’s perceptions of the acoustic environment (i.e., the soundscape) and analysing the sound environment from a positive perspective that transcends the restrictive pollution-related approach.

The key principles of soundscape are defined in international standard ISO 12913, in which the notion of soundscape is viewed as an acoustic analogy of landscape. ISO 12913:1:2014 provides the definition of a conceptual framework for the term soundscape [41], while ISO/TS 12913-2:2018 supplementary materials about data collection regarding soundscape studies [42]. According to this standard, soundscape is “the acoustic environment as perceived or experienced and/or understood by a person or people in context”.

But what are the beneficial effects of restorative environments on human health? According to Kaplan and Kaplan’s [1] and Ulrich’s [2,3,4] theories, natural settings reduce stress and alleviate negative emotional states, but there are few references to their impact on positive emotional states. Hence, San Juan et al. [19] have argued that urban design can also significantly contribute to improving people’s well-being and quality of life, reducing their stress and restoring their psychological state. Other studies on the soundscape have highlighted the benefits of sound environments for well-being, mainly focusing on natural [43] or human sounds [44]. Thus, several studies have tried to provide scientific evidence of the benefits of the “soundscape approach” for public engagement, health and well-being [45,46]. Consequently, the soundscape should be considered part of urban design [47,48], incorporating specific urban furniture [49] to improve people’s perceptions of urban places and their environmental experiences.

According to the WHO, “health is a state of complete physical, mental and social well-being rather than the absence of illness or discomfort” [50]. Hence, any analysis of restorative environments should consider both their capacity to mitigate negative health states (e.g., negative emotions, stress) and their benefits for positive health states (e.g., comfort, calm, happiness).

The literature shows that psychological restoration in natural settings has received considerable research attention in recent years, but such studies have focused mainly on the visual dimension. However, due to the global trend towards urbanisation, there is a need to extend these studies to urban settings, to integrate the dimension of sound into landscape, and to study the benefits for positive health states.

Therefore, the main objective of the present study was to determine the influence in urban open public spaces of the sound environment and its perception (soundscape) on users’ health. To this end, the effects on positive and negative emotional states and perceived stress were measured. A further aim was to identify the characteristics of the soundscape that enhance emotional restoration and reduce perceived stress.

Within the scope of this article, the authors consider urban open public spaces “as the total surface of the built-up areas of cities devoted to streets and boulevards—including walkways, sidewalks, and bicycle lanes—and the areas devoted to public parks, squares, recreational green areas, public playgrounds and open areas of public facilities” [51]. The present study focused on open public urban spaces whose primary function was to provide a leisure area, such as squares, parks or gardens.

The study hypothesis was that comfortable urban places with a pleasant soundscape are restorative in terms of their effect on emotions, complementing other studies on other restorative aspects, such as the physiological effects or considering their effect on attention, and should therefore be associated with an increase in positive emotions and a reduction in negative emotions such as perceived stress.

## 2. Materials and Methods

**Figure 1:**
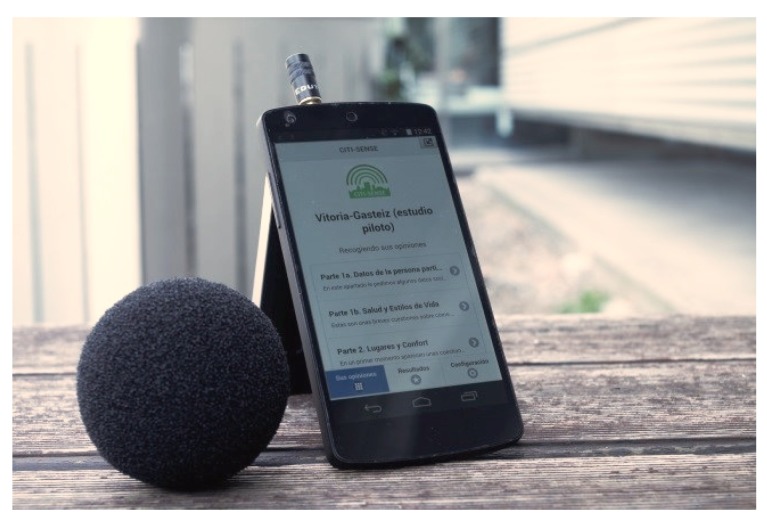
CITI-SENSE observation toolkit.

The present study was conducted using a toolkit developed as part of the CITI-SENSE EU Project [52,53] (for more information, visit the project web site www.citi-sense.eu.) which allowed a simultaneous assessment of the acoustic environment and its perception (soundscape) on site. This toolkit [37] is an adapted smartphone and app designed to facilitate observations of open spaces (see Figure 1).

It comprises five elements: (a) a smartphone, which can be used to post-process acoustic signals; (b) an external microphone for measuring acoustic levels; (c) a user-friendly smartphone app that enables people to provide an assessment and collects data on their perceptions of the area via an embedded questionnaire; (d) a procedure for measuring the acoustic environment and soundscape, based on the state-of-the-art; and (e) a protocol for conducting observations that includes clear instructions for participants.

As explained above, the present study focused on open public urban spaces whose primary function was to provide a leisure area, such as squares, parks or gardens. These places were selected because people visit them to rest, relax or socialise, activities that are closely associated with restorative environments and which also define the functions of urban open public spaces. Hence, if these types of urban places are pleasant, they are more likely to have a restorative capacity because people will spend more time enjoying them. The combination of the characteristics of these places and people’s perceptions and enjoyment of them determines environmental and acoustic comfort. The public spaces analysed in this study will henceforth be referred to as “urban places”.

Other types of urban public space, such as streets, stations or enclosed spaces, were not included in the study because the main function of the first is to provide a place of transit, while the latter two usually offer very little contact with natural elements related to vegetation (green) or water (blue).

### 2.1. Case Studies

**Figure 2:**
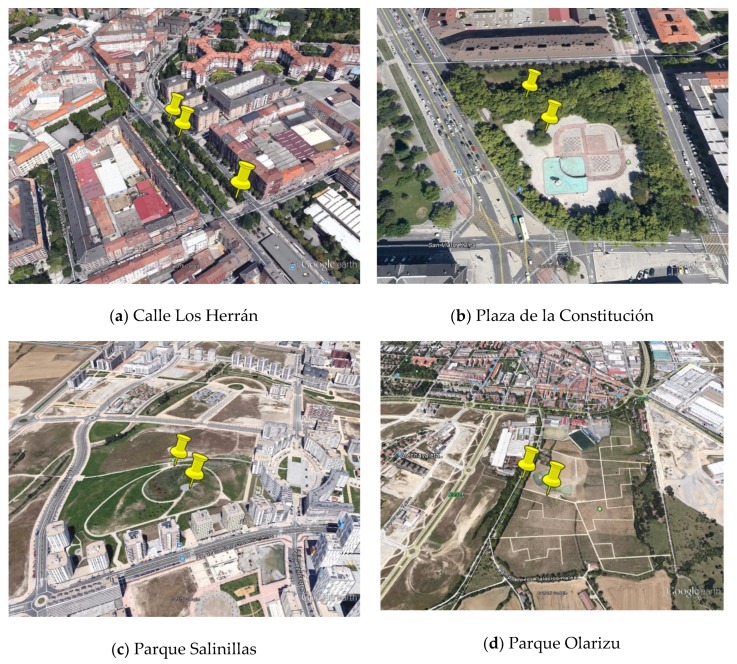
Pictures of the four urban places, indicating the evaluation points. Source: Google Earth.

Four urban places in the city of Vitoria-Gasteiz (Spain) were selected to carry out the study. Selection was conducted with the aim of obtaining a sample of places with diverse characteristics in terms of the presence of natural elements (green and blue) and their function in the city (frequently used and located in the city centre or more sporadically used and located in a peri-urban environment). The places selected were (Figure 2):

- Los Herrán (Figure 2a). Stretch of the Los Herrán street in which the city’s central bus station was previously located. The place analysed in this street is the central area with leisure use (boulevard), which is surrounded by high traffic flow roads and is close to a school.
- Constitución (Figure 2b). Constitución square is situated next to the northern entrance to the city. To the left of the square, there is a relatively quiet green street.
- Salinillas (Figure 2c). Salinillas de Buradón park is situated in a new urban area and sits on a small hill close to the city’s green belt. The park has very few trees.
- Olarizu (Figure 2d). Olarizu park is part of the city’s green belt and home to the Environmental Research Centre (CEA), which receives thousands of visitors throughout the year. Some of these spend the day in the surrounding area.

The method proposed to characterise each urban place comprised an assessment of a set of objective variables related to the quality of services and the diversity of each place, the presence of green (green), cultural (landmarks and heritage) and water (blue) elements, the level of artificiality (grey) and the proportion of openness (% sky) [37].

The maintenance, safety, presence of businesses (shops), traffic, facilities and tall vegetation (trees) were also evaluated. These were assessed on a scale of 0–3 (0 not applicable; 1 low; 2 medium; 3 high). In addition, water, landmarks and heritage were assessed on a scale of 0–2 (0 not present; 1 yes, it can be seen from the study area; 2 yes, it is part of the study area). These evaluations were based on the ratings of three project technicians and are the result of a consensus process (Delphi Method). More detailed information on this evaluation methodology is available in QUADMAP guidelines [54].

Table 1 provides a description of the four urban places analysed in relation to their physical and landscape features.

The four places displayed a great deal of homogeneity in terms of maintenance, which was generally high, the presence of water, landmarks and heritage. In contrast, they differed widely in terms of safety, facilities, traffic, economic activity, trees and green areas. These differences were also reflected in characterisation of the places in terms of greenness (% green), water (% blue), artificiality (% grey) and openness (% sky).

When the places were ranked according to the results of this analysis, a dichotomous dimension emerged: grey versus green (or artificiality versus naturalness). Grey was defined by building elements and artificiality, such as the presence of shops, traffic and facilities (urbanisation), whereas green was defined by the presence of vegetation, greenness and water (naturalness). The four places analysed in the study were ranked as follows according to this dimension: Los Herrán, the greyest place; Constitución, a grey place; Salinillas, a green place with sparse vegetation; and Olarizu, the greenest place with water.

### 2.2. Procedure and Data Collection

A protocol was established to define how participants should conduct their acoustic observations of the urban places. Since the observational procedure was both crucial and complex, participants were accompanied by a member of the team who guided them in order to ensure that it was applied correctly during the exercise. At the beginning, participants spent five minutes observing the location to gain experience of the urban place (urban environmental experience), as they were expected to make conscious observations and assessments. Sound events could occur at any time during the observation, and each time one was detected, a pop-up message was displayed on the smartphone screen. The message prompted users to identify their perception (i.e., pleasant or unpleasant) and the type of acoustic source for the event. Participants identified the main acoustic sources noticed, but without identifying the potential keynote sounds of each urban place. As soon as the evaluation was completed, data were post-processed, providing observers with easily interpretable feedback on their evaluation.

The observations were conducted from the 17th to the 30th of April, 2015. It was important for this study that the weather facilitated enjoyment of the urban places analysed; consequently, data collection was conducted on sunny spring days when it was neither very cold nor very hot and participants were available. Experiences were usually collected at times when the spaces were most crowded (10am–1pm and 5–8pm). The mean duration of experiences was 12.45 min (SD = 6.76), with no significant differences between places.

The acoustic indicators were measured for the duration of the observer’s experience in these places.

Data protection legislation and participants’ rights and obligations with respect to the data they collected were observed at all times. To fulfil the legal requirements of the European Directive 95/46/EC, 24th October 1995 on the protection of individuals with regard to the processing of personal data and on the free movement of such data, and Spanish Law 15/1999, 13rd December, of Protection of Personal Data) a privacy policy document was drafted. It described the type of data to be collected, its intended use (e.g., for research and scientific publications), data storage and protection. All these details were gathered in two documents signed by users/participants (i.e., the Privacy Policy and the User Agreement) [55].

### 2.3. Sample

Participation was voluntary, and subjects were recruited from among residents of the city of Vitoria-Gasteiz, through civic associations. The criteria for selecting participants were established by the Iritziak Batuz team, and the process is described in deliverable D3.4 of the CITI-SENSE project [56]. A total of 53 people conducted field observations in the four urban places. They produced a total of 137 observations that were used for analysis, and each participant evaluated at least two sites in the same or in different urban places. In this regard, the unit of analysis for this study was each of these 137 observations.

### 2.4. Assessment of Sound Environment

Preliminary tests under environmental conditions indicated that the smartphone’s built-in microphone was highly sensitive to wind (contribution higher than 5 dB with wind intensity above 1.5 m/s), which would have affected outdoor measurements. Therefore, an external microphone with a standard protective windscreen was added to the measurement protocol. After analysis and a search for a low-cost microphone, the Edutige EIM-003 was chosen. The improvement in accuracy was tested in an anechoic chamber, and it was found that the average deviations of 6.7 dB (obtained using the smartphone with an internal microphone) were reduced to 1 dB (obtained using the smartphone with an external microphone), compared using a class 1 sound level meter.

The toolkit was designed to measure global LAeq,1s levels, as other acoustic indicators can be constructed from this parameter: LAeq,T, minimum LAeq,1s as LAmin and maximum LAeq,1s as LAmax. As part of the measurement, the time history is registered and displayed on the smartphone screen, as is the global mean LAeq,T level and the maximum and minimum LAeq,1s levels during the measurement period. In addition, the toolkit detects sound events by applying a dynamic threshold principle, and when an event is detected, the participant is prompted to provide an assessment (e.g., pleasantness and type of sound source).

This method is based on a comparison of the instantaneous LAeq,1s level with the energy means of the LAeq,5s, downstream (5 s earlier) and LAeq,5s, upstream (5 s later). Thus, a sound event is detected when the acoustic level variation indicates a difference with both downstream and upstream means that exceeds the threshold (6.5 dBA fixed value). The fixed threshold value was defined using expertise to identify events in noisy or quiet urban environments alike.

### 2.5. CITI-SENSE Questionnaire: Emotions, Soundscape and Other Issues

Although the CITI-SENSE toolkit allows a simultaneous assessment of the sound environment and collection of environmental perceptions on site, participants’ responses to the questionnaire were independent of sound or acoustic environment measurements. The questionnaire was only interrupted when an event was detected during the two minutes that the acoustic environment was being measured, to ask participants what kind of event it was and whether or not they found it pleasant.

The CITI-SENSE questionnaire, which can be consulted in the Supplementary Materials, collected information on participants’ emotional states, the soundscape and other variables that might also influence emotional state.

Emotional impact was evaluated using an emotions scale that included four basic emotions, two of which were positive; happiness (high arousal) and calm (low arousal), and two negatives; anger (high arousal) and sadness (low arousal). The five-point scale also included an item to measure perceived stress. These five items were assessed at two different times, once at the beginning of the questionnaire (referring to emotional states in the preceding days), and subsequently at the end of the questionnaire (referring to present emotional state after urban environmental experience). Differences in the scale between these two moments indicated the emotional impact of urban environmental experiences in the places analysed.

Soundscape (SSC) was evaluated by means of an ad hoc questionnaire, using a semantic differential (SD) scale that contained 13 pairs of bipolar adjectives such as unpleasant-pleasant, noisy-calm, stressful-relaxing, artificial-natural, monotonous-lively (vibrant), informative-uninformative and inappropriate-appropriate to surroundings, rated using a five-point ordinal scale. The data collection method corresponded to that described in ISO/TS 12913:2 on soundscape [42].

A semantic differential five-point scale was also used for landscape (LSC), with 3 items related to unpleasant-pleasantness, noisy-quietness and artificial-naturalness. These items did not specifically include visual aspects.

In addition, other aspects were considered that might influence the relationship between soundscape and its emotional impact. These included sociodemographic variables, residential factors, general self-perceptions of health and acoustic and environmental comfort.

Acoustic and environmental comfort were evaluated by means of two specific items measured on a 5-point ordinal scale (where 1 = very uncomfortable and 5 = very comfortable). The scale also included assessments of thermal, lighting and visual comfort, which were not specifically analysed in the present study.

### 2.6. Data AnalysisStrategy

To describe the data, the mean and standard deviation (M± SD) were calculated in the case of continuous variables, and the frequency and percentage (n, %) in the case of nominal variables. The contrast of proportions was performed through the Chi-Square test (χ^2^) or the equivalent Fisher’s exact test in the case of expected frequencies less than five. For the contrast of mean differences, the analysis of variance test (ANOVA) was used, and in the case of non-compliance with the homoscedasticity assumption, the Brown-Forsythe robust test was applied. Normal distribution was also checked prior to contrast analysis using the Kolmogorov-Smirnov test. The degree of association between variables was estimated using Pearson’s (r) product-moment correlation coefficient. Likewise, multiple linear regression models were used to determine the predictors of the emotional response, with an estimation of the validity of the model (ANOVA test), the level of variance explained by the retained factors (R^2^) and an estimation of the standardised coefficient (β) of each of them. For all analyses, the level of significance considered was α = 0.05.

## 3. Results

The results obtained for the proposed solution as regards the research objectives are presented below and include: (1) characterisation of study participants; (2) characterisation of the urban places: acoustic levels and soundscape; (3) emotional effect of the urban environmental experience (henceforth urban experience); (4) explanation of the emotional effect of urban environmental experiences (UEE).

### 3.1. Characterisation of Study Participants

Women accounted for 54% of the study sample and men, 46%. The mean age of participants was 42.3 years (SD = 14.18 years, min = 19; max = 75). In addition, 46.4% had a university and 39.0% a secondary education, and 40.4% were employed while 16.9% were unemployed.

As shown in Table 2, in general, there were no significant social or demographic differences between the observers in each of the urban places. The only significant difference concerned place of residence (χ^2^ = 28.263; df = 15; *p* < 0.05), whereby residents of Vitoria-Gasteiz accounted for all (100%) of the participants who assessed Olarizu but only 80.7% of those who assessed Constitución square.

Neither was a statistically significant difference between urban places and participants’ perceived health status, which was generally good (51.1%) or very good (27%). No participants perceived their health status to be bad.

These results indicate that composition of the participant group did not affect the results of the analysis.

### 3.2. Characterisation of the Urban Places: Sound Environment and Soundscape

The urban places with the highest acoustic levels were Los Herrán and Constitución, at 60.9 and 60.5 dBA LAeq, mean, respectively (Table 3). Los Herrán was also where the highest maximum level was recorded (79.3 dBA maximum-LAeq,1s), whereas the highest minimum level was recorded at Constitución (51.9 dBA minimum-LAeq,1s). Acoustic events were barely detected at these places.

Participants were asked to identify the most characteristic sound sources in the urban places and the pleasantness of their sound environments. The most common source of sound was traffic (64.3%) at Los Herrán, natural sounds (29.0%) and traffic (25.8%) at Constitución and sounds associated with nature at the greener places, Olarizu and Salinillas (93.3% and 55.0%, respectively) (χ^2^ = 89.81; df = 18; *p* < 0.001). Thus, the main sound source at Los Herrán, the most artificial place, was considered unpleasant (85.7%), while at Olarizu, the greenest place, it was considered pleasant (93.3%), and these differences were statistically significant (χ^2^ = 78.26; df = 12; *p* < 0.001). In relation to the above, it can be seen that the number of acoustic events (mostly positive) was higher in the greener than in the greyer place.

Acoustic and environmental comfort (assessed using a 5-point ordinal scale) was high in Olarizu (4.0 and 4.1, respectively), medium-high in Salinillas (3.8 and 3.6), average in Constitución (2.9 and 3.3) and low (2.2) or medium-low (2.6) in Los Herrán, and all of these differences were statistically significant (F(3,133) = 37.92; *p* < 0.001 and F(3,133) = 21.78; *p* < 0.001).

The results for soundscape (SSC) characterisation were similar to those for comfort, as can be seen in Table 3. A semantic differential five-point scale with bipolar adjectives was used to assess the soundscape; however, the table only gives the right-hand adjective of each pair, which corresponds to the highest value (5) for the response options.

Soundscapes at Olarizu were generally associated with positive scores (mean values around 4, on a scale of 1–5), with the exception of the score for lively, which was neutral (3.2). Overall, the Olarizu soundscape was characterised by naturalness (4.5) and capacity to facilitate conversation (4.3).

The Salinillas soundscapes were associated with scores between neutral and positive (values between 3 and 4). Meanwhile, at Constitución, they were associated with neutral scores (around 3), scoring higher for familiarity (4.0), appropriacy to the environment (3.6) and uninterrupted (3.5).

The soundscapes at Los Herrán obtained the lowest scores, especially for lack of calm (1.7), naturalness or pleasantness (1.9). As with Constitución, its soundscapes were also familiar (4.0) and uninterrupted (3.8).

Perceptions of the degree of naturalness of the landscape in each urban place confirmed the previous ranking of these according to the dimension of grey versus green. Thus, Olarizu, the urban place ranked as the most natural because it contained the most green and blue elements (Table 1), was the one that participants perceived as the most natural (mean 4.53 in LSC-natural: Table 3), whereas Los Herrán, the most artificial place (85% grey in Table 1) with the least green elements (25%) was perceived as the most artificial (mean 2.36 in LSC_natural: Table 3).

### 3.3. Emotional Effect of the Urban Environmental Experience

**Figure 3:**
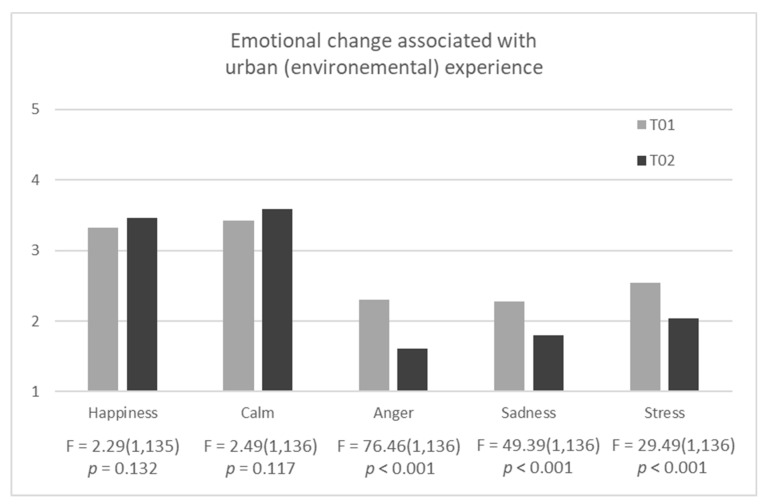
Effect of the urban experience on the four basic emotions and perceived stress, before (T01) and after (T02) renovation of the urban places (ANOVA test).

Environmental experiences in the four urban places analysed in Vitoria-Gasteiz gave rise to emotional changes, as can be seen in Figure 3, which gives the mean scores of the total number of observations, for each of the four basic emotions considered and perceived stress at the beginning (T01) and end (T02) of the urban experiences.

The changes indicate that after the urban environmental experience, even if this had been of a short duration, positive emotions increased slightly, and negative emotions and perceived stress were reduced. A repeated measure analysis revealed that differences in the positive emotions of happiness and calm were not statistically significant. In contrast, reductions in negative emotions and perceived stress were statistically significant. Anger dropped by 0.69 points (30%) (F(1,136) = 76.493; *p* < 0.001), sadness by 0.49 (21.55%) (F(1,136) = 49.386; *p* < 0.001) and perceived stress by 0.50 (19.7%) (F(1,136) = 29.493; *p* < 0.001). The effect size (partial Eta squared: η^2^) was large in all three cases (0.360 for anger, 0.266 for sadness and 0.178 for perceived stress).

Table 4 presents the mean scores for emotional states before and after the urban experiences, and the difference between both, for the total number of observations and for the places analysed. As can be seen in the table, apparent differences emerged between the urban places, although these did not reach statistical significance.

These differences (Table 4) suggest that at Los Herrán, the least green place, the urban experiences did not change positive emotions. However, they reduced negative ones and perceived stress, although to a lesser extent than in the other urban places. In Los Herrán, perceived stress was greater at the end of the urban experience, presenting tendential differences to the other urban places (F(3,133) = 2.40; *p* = 0.07).

Urban experiences in the second least green place, Constitución, were not associated with changes in positive emotions either, but again, they did reduce negative ones and perceived stress, even to a slightly greater extent than experiences in the parks of Salinillas and Olarizu, the greener places. Urban experiences in these latter were associated with a slight increase in positive emotions and a reduction in negative emotions, mainly anger, and perceived stress (Table 4).

Table 5 shows the relationships (Pearson correlation) between the dimensions of environmental and acoustic comfort and emotional states at the end of the urban experience. As can be seen, comfort was inversely associated with negative emotional states (as the former increased, the latter decreased), whereas it presented a direct relationship with positive emotional states (as the former increased, so did the latter).

Thus, happiness in the urban settings was directly related to perceptions that the soundscape was calm and pleasant and to the environmental comfort of the urban environmental experience (UEE) (*r* ≈ 0.40). It was also associated—albeit less strongly—with acoustic comfort and the fun and naturalness of the soundscape (*r* ≈ 0.40). Calm at the end of the urban experience was also related to environmental and acoustic comfort and to perceptions of pleasantness, calm, fun and naturalness of the soundscape (*r* ≈ 0.25. As with happiness, perceived stress also presented a strong association with environmental and acoustic comfort and with pleasantness of the soundscape, but this time inversely (r ≈ –0.40).

Similarly, the negative emotions of anger and sadness were inversely associated with environmental and acoustic comfort and the pleasantness of the soundscape (correlations between –0.2 and –0.3). Sadness was also inversely related to the liveliness or vibrancy and naturalness of soundscapes; thus, the livelier and more natural the soundscape, the lower the sadness at the end of the urban experiences.

### 3.4. Explaining the Emotional Effect Of Urban Environmental Experiences

To determine the explanatory power of the factors considered, mainly those related to the soundscape and the sound environment, for emotional effect and perceived stress, multiple regression models were constructed.

Having selected four basic emotions (happiness, calm, anger and sadness) and perceived stress as outcome variables, five regression models were constructed using the same set of independent variables: sociodemographic variables (e.g., age, sex and educational level), the parameters used to characterise the acoustic environment of the urban places analysed (LAeq,mean, min-LAeq,1s, max-LAeq,1s, no. of positive, negative and total events, and pleasantness of the two main sound sources), acoustic and environmental comfort of the places and soundscape (13 dimensions considered in the semantic differential scale). To control for the effect of participants’ emotional state when commencing the experience, baseline measurements of the five emotional variables were included in the regression model. Each model was constructed using a stepwise strategy whereby those factors that did not significantly explain the emotion assessed were gradually discarded.

Table 6 shows the five regression models, one per column, together with the predictive variables that were statistically significant in one of the models. The predictive model for the emotion of calm was the most modest, explaining 22.5% of the variance (R^2^ = 0.225) and identifying three predictors, whereas the predictive model for stress was the most robust (R^2^ = 0.519).

It should be noted that in the social sciences, the average value of associations between variables is around 0.21 [56]; therefore, the correlation value obtained in the present study was close to this average and even a little above.

An analysis of the data presented in the table for stress indicated that this model was statistically significant (F = 25.11; *p* < 0.001) and identified six predictors of stress: experiencing low environmental comfort (*β* = −0.34), the presence of high baseline anger (*β* = 0.33), perceptions of the soundscape as lively or vibrant (*β* = 0.29), the presence of baseline sadness (*β* = 0.23), a lower educational level (*β* = −0.21) and a high baseline level of stress (*β* = 0.19).

Focusing on the factors involved in the expression of emotional states, the results can be assessed by analysing how many of the emotions presented a given explanatory factor. In this regard, for example, a baseline emotion of anger was associated with four of the emotional states: negatively with happiness (*β* = −0.20) and positively with anger (*β* = 0.45), stress (*β* = 0.33) and sadness (*β* = 0.20). Meanwhile, environmental comfort was associated with greater happiness (*β* = 0.27) and less stress (*β* = −0.34) or anger (*β* = −0.26).

These results indicate that emotional states after the urban experience were generally associated with previous emotional states, but primarily with negative emotions and perceived stress.

For the acoustic environment, only the LAeq, mean was significantly associated with the emotional states of calm and sadness after the urban experience. Higher sound levels were associated with emotional states of greater calm and less sadness.

The environmental experiences in more comfortable urban places were associated with greater happiness and less anger or perceived stress.

With regard to the soundscape, differences were observed according to the emotion considered. Calm was associated with acoustic comfort, while in contrast, the negative emotion of anger was positively associated with the soundscape’s capacity for fun: the more fun a soundscape was considered, the more anger the observer felt at the end of the experience. Similarly, the more lively or vibrant the sound environment was perceived to be, the more stressed the observer felt at the end of the urban experience.

## 4. Discussion

In agreement with other studies [6,10,11,14,19,20], the results reported here confirm the initial study hypothesis that some urban places exert a positive impact on people’s well–being and quality of life. Thus, the present study has demonstrated a positive effect that reflects the emotionally restorative capacity of the urban places analysed, whereby environmental experiences in these places yielded a statistically significant reduction in perceived stress and the negative emotions of sadness and anger, and a trend towards an increase in the positive emotions of happiness and calm.

In this study, the effect on positive emotions did not reach statistical significance. This may be because these emotional states are more resistant to change and require visits of a longer duration to reflect these benefits; recall that the duration of the urban environmental experiences in this study was short. A further possibility is that the beneficial effect on positive emotions is more strongly associated with the naturalness of places [1,2,3,4,5] and it is therefore more difficult to find this effect in urban environments. Another possible explanation may be the paradox whereby it is difficult to improve situations that are already close to their maximum. In general, when people are asked about positive emotional states, health, life satisfaction or residential surroundings, they tend to respond positively and close to the maximum, leaving little room for improvement. As with all horizontal asymptotes, the value of “y” will never be equal, and it can always be improved and brought closer to the maximum; however, in practice, the difference is imperceptible and sometimes even irrelevant. It might therefore be more difficult to improve positive emotions than negative ones. The same occurs in other areas such as athletic performance or life expectancy, which evidence increasingly smaller improvements.

The results presented here also indicate that the emotionally restorative capacity of the urban places was influenced by the environmental comfort experienced by the participants. Evidently, emotional states, especially negative ones, at the end of the urban environmental experiences were to a large extent determined by prior emotional states. However, besides these, the other aspect that exerted the most influence on participants’ emotional states at the end of the experience was environmental comfort, conceived holistically (globally). These results highlight the need for a holistic vision of experiences of places in urban environments, in order to integrate information from all the senses.

Besides global environmental comfort, acoustic comfort was also associated with a reduction in negative and an increase in positive emotional states. This result is in agreement with recent studies analysing the relationship between soundscape and health [57] and the restorative capacity of the soundscape in urban settings e.g., [33,34]. In this respect, this work complements other studies aimed at improving physiological and attention restoration [1,2,10,14]. This study found that the soundscape characteristics which contributed to greater emotional restoration and a reduction in perceived stress were pleasantness, calm, fun and naturalness. It therefore contributes to analyses of the soundscape characteristics that can exert positive health-related effects. A recently published systematic review of associations between positive soundscapes (e.g., pleasant, calm, less annoying) and health (e.g., increased restoration, reduced stress-inducing mechanisms) found that positive soundscapes are associated with faster stress recovery processes in laboratory experiments and better self-reported health in large-scale surveys [46]. Hence, the present study adds to the above-mentioned research by providing results obtained using a method applied in real urban environments.

Although this study was conducted on site, the method applied made it possible to collect objective and subjective data simultaneously; consequently, the objective acoustic environment indicators referred specifically to the period of time in which each participant’s urban environmental experience took place. In this respect, the present study supports the use of the new technologies, a smartphone application in this case, to conduct soundscape research based on subjective data reported directly by users of the places. However, the application of this method proved somewhat complex and might present limitations compared to laboratory studies, as might the study sample size and the low diversity of urban spaces have considered. It is also possible that the experiment itself or the attention the participants received contributed to the effect on emotional restoration. Nevertheless, the method applied has proved interesting, and the study findings, while not conclusive, are consistent with the results obtained in earlier studies employing other research methods.

**Figure 4:**
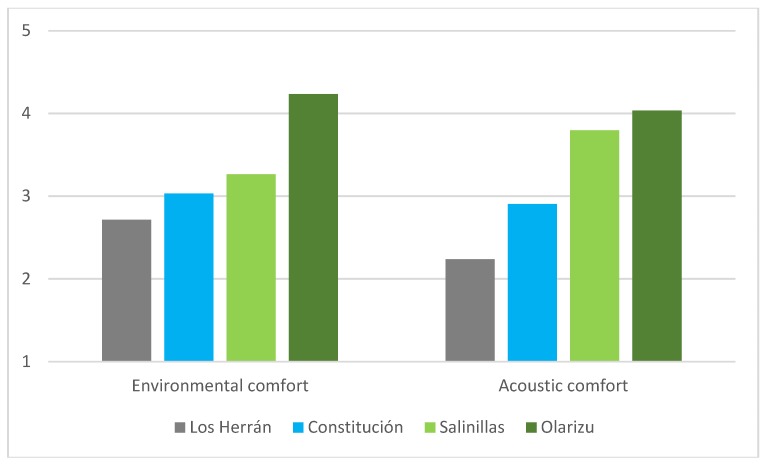
Mean scores for environmental and acoustic comfort in relation to the artificial-natural continuum.

In addition, in this study, environmental comfort (both global and acoustic) presented an increase associated with the dichotomous dimension of artificiality (% grey elements) versus naturalness (% green and blue), as shown in Figure 4. The greener the places, the more environmentally and acoustically comfortable they were.

The analysis of acoustic environments and their perception (soundscape) indicates that besides naturalness, sound diversity was associated with greater pleasantness and comfort. Acoustic environments in more artificial places were characterised by higher acoustic levels, meaning that fewer sound events were detected. In contrast, the acoustic environment of spaces with a high presence of natural elements presented lower acoustic levels and a higher number of events, which tended to be perceived positively. In addition, the most characteristic sound sources in these environments, usually human or natural, were considered pleasant. Participants were not asked to identify potential keynote sounds, and therefore it was not possible to analyse whether these contributed to the acoustic comfort or restorative capacity of urban open public spaces.

The classical theories of attention restoration—ART [1] and stress recovery, SRT [2,3,4]—state that there is a direct link between naturalness and restorative capacity. Consequently, and given the relationship between comfort and naturalness detected in the present study, it would seem logical to conclude that naturalness influences emotional restoration. However, no evidence of a direct association between the two was obtained in the present study, since no significant differences were observed between the urban places for any of the emotions analysed. This would seem to indicate an indirect relationship mediated by the environmental comfort experienced by users in the place.

The results evidence some limitations since it has not been possible to provide very strong evidence of the restorative capacity of urban open public spaces or to quantify the relationship between restorative capacity and the presence of natural elements in urban places. Nevertheless, the study has provided indications in the expected direction. This suggests the need to continue to conduct research in this area, which encompasses two topics for which previous studies have reported firm evidence, namely the restorative capacity of some urban settings and the contribution of the soundscape to this positive effect on health and well-being.

This work complements other studies [10], as it is focused on small urban public spaces. In future research, it would be interesting to extend the diversity of urban open public spaces in order to further elucidate the contribution of different elements: green (e.g., grass, shrubs, trees), blue (e.g., fountains, rivers, lakes, seas), and grey (e.g., types of material, design). It would also be interesting in future research to consider cultural diversity (mainly related to cultural heritage and signs of identity), social diversity (related to users) and animal biodiversity (the activity of which could contribute to increase the richness of the associated soundscape).

The findings of this study, although not conclusive, give evidence of the potential use of this method to analyse the restorative effect of the quality of urban public spaces, and their soundscape, on emotions. In combination with ICT technologies, it can enable studies where citizens can take part as users of the spaces, reducing their participation effort. As an example of this, the observation time necessary in this survey was around 20 minutes, lower than that needed in other studies [10,14,19,20]. Consequently, the completion of field studies was much easier and the outcomes showed that emotional restorative effects could be noticed with short periods of environmental experience. Nonetheless, the influence of the length of time of the test should be investigated in greater depth, considering the target restorative effect to be assessed (physiological, attention, emotions or other). As other authors have noted, it is “necessary to plan and conducive to the soundscape and health research within the larger framework of environmental quality and life quality, restoration, coping, and environmental and social health” [45].

## 5. Conclusions

The main conclusion that can be drawn from this study is that the capacity for psychological restoration is not unique to natural settings outside cities, but may also be a characteristic of some urban spaces, since it was found that even for short periods of time, the use of urban places was associated with a significant decrease in negative emotions and perceived stress, as well as a slight increase in positive emotions.

Another important aspect is that restorative capacity is associated with global environmental comfort and acoustic comfort in particular. Thus, the higher the comfort, the better the emotional state detected. Consequently, acoustically comfortable urban places with a pleasant soundscape can be considered restorative environments.

Since the urban soundscape can promote the psychological restoration of users, it should clearly form part of planning and architectural design [47,58], incorporating specific urban furniture [49] to improve the perception of urban places. In this respect, collaboration from the initial stages of project development between those responsible for urban design and acoustics experts represents a crucial element in urban renovation processes [59]. The human and social sciences should also play a key role in holistic soundscape studies because soundscape is a construct of human perception.

This study identified the soundscape characteristics that contributed to greater positive health-related effects, namely pleasantness and calm, as well as the attributes of fun and naturalness. These findings confirm the known benefits of enhancing the natural component of urban places and increasing their acoustic diversity, for example by encouraging insects and birds, facilitating the interaction between wind and plants, or introducing the sound of water. In addition, designing and creating other positive acoustic events and sound sources would also contribute to improving environmental experiences in these urban places.

## Figures and Tables

**Table 1:** Description of the four urban places selected.

**Evaluation (1)**	**Los Herrán**	**Constitución**	**Salinillas**	**Olarizu**
Maintenance	3	3	2	3
Safety	2	3	1	2
Shops	2	1	0	1
Traffic	3	1	1	1
Facilities	2	2	0	1
Trees	2	2	1	2
**Presence (2)**	**Los Herrán**	**Constitución**	**Salinillas**	**Olarizu**
Water	0	2	0	2
Landmarks	0	0	0	1
Heritage	0	1	0	0
**Percentage**	**Los Herrán**	**Constitución**	**Salinillas**	**Olarizu**
% green	25	40	90	80
% blue	0	25	0	25
% sky	35	30	100	80
% grey	85	60	10	10

(1) Evaluation: 1 low; 2 medium; 3 high; 0 not applicable. (2) Presence: 0 no; 1 yes, it can be seen from the study area; 2 yes, it is part of the study area.

**Table 2:** Participant characteristics by urban places in Vitoria-Gasteiz (total), and significance (*p*) of Chi-square analysis.

No. of Observations	Los Herrán	Constitución	Salinillas	Olarizu	% GLOBAL		
	42	31	34	30	137	*N*	*p*
Gender (women)	52.4%	51.6%	61.8%	50.0%	54.0%	74	*0.769*
Residence (Vitoria-Gasteiz)	92.9%	80.7%	94.1%	100.0%	92.0%	126	0.020
University education	43.9%	45.2%	50.0%	46.7%	46.3%	63	*0.885*
Secondary education	46.3%	32.3%	35.3%	40.0%	39.0%	53	
Primary education	4.9%	12.9%	8.8%	10.0%	8.8%	12	
Other	4.9%	9.7%	5.9%	3.3%	5.9%	8	
Employed	36.6%	45.2%	41.2%	40.0%	40.4%	55	*0.069*
Unemployed	14.6%	6.5%	20.6%	26.7%	16.9%	23	
Students	29.3%	12.9%	8.8%	0.0%	14.0%	19	
Retirees	7.3%	16.1%	5.9%	10.0%	9.6%	13	
Other	12.2%	19.4%	23.5%	23.3%	19.1%	26	
Perceived health: good and very good	76.1%	71.0%	82.4%	83.4%	78.1%	107	*0.605*
P. Health: fair	23.9%	29.0%	17.6%	16.6%	21.9%	30	
P. Health: bad	0.0%	0.0%	0.0%	0.0%	0.0%	0	

N: frequency of response by category; p (probability of χ2): * *p* < 0.05; ns: not significant.

**Table 3:** Characterisation of the urban places analysed (mean scores ± standard deviation), by sound environment, landscape, environmental comfort and soundscape, and significance (*p*) of ANOVA analysis.

N	Los Herrán	Constitución	Salinillas	Olarizu	Global	*p*
	42	31	34	30	137	
dB LAeq,mean	60.9 ± 4.21	60.5 ± 10.38	52.9 ± 16.63	55.9 ± 8.92	57.7 ± 9.78	*0.001*
dB max LAeq,1s	79.3 ± 6.09	74.7 ± 10.65	76.3 ± 15.54	74.7 ± 9.46	76.4 ± 10.83	*0.261*
dB minLAeq,1s	47.6 ± 3.36	51.9 ± 6.21	43.8 ± 11.81	43.4 ± 6.83	46.7 ± 8.13	*<0.001*
No. total events	1.41 ± 1.97	1.10 ± 9.80	7.98 ± 1.19	8.23 ± 7.02	4.49 ± 6.87	*<0.001*
No. positive events	0.53 ± 0.88	0.77 ± 5.57	4.91 ± 1.07	5.31 ± 4.92	2.73 ± 4.33	*<0.001*
No. negative events	0.80 ± 1.19	0.29 ± 3.60	1.94 ± 0.59	2.57 ± 4.47	1.36 ± 2.95	*0.007*
LSC_pleasant	2.71 ± 1.07	3.74 ± 0.77	3.7 6± 0.99	4.5 3± 0.73	3.61 ± 1.13	*<0.001*
LSC_quiet	1.98 ± 1.07	3.32 ± 1.19	4.03 ± 0.87	4.20 ± 0.71	3.28 ± 1.34	*<0.001*
LSC_natural	2.36 ± 1.01	2.61 ± 0.99	3.32 ± 1.12	4.53 ± 0.63	3.13 ± 1.26	*<0.001*
Environmental comfort	2.60 ± 0.80	3.32 ± 0.96	3.56 ± 0.75	4.10 ± 0.66	3.33 ± 0.97	*<0.001*
Acoustic comfort	2.24 ± 0.82	2.90 ± 0.84	3.79 ± 0.83	4.03 ± 0.72	3.17 ± 1.09	*<0.001*
SSC_pleasant	1.90 ± 0.88	2.97 ± 0.93	3.53 ± 1.05	4.10 ± 0.66	3.03 ± 1.22	*<0.001*
SSC_calm	1.69 ± 0.84	2.90 ± 0.99	3.62 ± 1.04	3.97 ± 0.76	2.94 ± 1.28	*<0.001*
SSC_relaxing	2.24 ± 0,91	3.23 ± 0.83	3.82 ± 0.76	4.10 ± 0.71	3.26 ± 1.10	*<0.001*
SSC_uninterrupted	3.79 ± 0.95	3.48 ± 0.86	3.47 ± 0.89	3.67 ± 1.03	3.61 ± 0.93	*0.407*
SSC_familiar	4.02 ± 0.90	4.03 ± 1.08	3.50 ± 0.84	4.10 ± 0.80	3.91 ± 0.94	0.029
SSC_facilitates conversation	2.48 ± 0.83	3.23 ± 0.88	3.88 ± 0.76	4.33 ± 0.66	3.40 ± 1.07	*<0.001*
SSC_informative	2.48 ± 0.89	2.87 ± 0.74	3.24 ± 0.76	3.23 ± 1.10	2.92 ± 0.93	*0.001*
SSC_clear	2.57 ± 1.15	3.19 ± 0.73	3.79 ± 0.86	4.03 ± 0.81	3.34 ± 1.09	*<0.001*
SC_characteristic	2.36 ± 1.32	2.90 ± 0.94	3.29 ± 1.16	3.90 ± 0.96	3.05 ± 1.25	*<0.001*
SSC_lively (vibrant)	2.60 ± 1.15	2.77 ± 0.98	3.21 ± 0.88	3.17 ± 0.99	2.91 ± 1.04	*0.028*
SSC_fun	2.43 ± 0.83	2.71 ± 1.03	3.09 ± 0.97	3.67 ± 0.92	2.93 ± 1.03	*<0.001*
SSC_natural	1.90 ± 0.96	2.58 ± 1.32	3.32 ± 1.12	4.40 ± 0.67	2.96 ± 1.39	*<0.001*
SSC_appropriate-surroundings	3.10 ± 1.14	3.58 ± 1.13	3.62 ± 0.81	4.20 ± 0.66	3.58 ± 1.05	*<0.001*

SSC: soundscape; LSC: landscape; SSC, LSC and comfort: 5-point scales; *p*: probability.

**Table 4:** Characterisation of emotional states at the beginning (T01) and end (T02) of the urban experience, and the difference between both (mean scores ± standard deviation) in the four urban places, and significance (*p*) of ANOVA analysis.

N	Los Herrán	Constitución	Salinillas	Olarizu	Global	*p*
	42	31	34	30	137	
Happiness_T01	3.29 ± 1.04	3.35 ± 1.07	3.21 ± 1.14	3.47 ± 0.90	3.32 ± 1.04	*0.782*
Happiness_T02	3.29 ± 0.74	3.48 ± 0.97	3.45 ± 0.81	3.70 ± 0.75	3.46 ± 0.82	*0.218*
Happiness_Dif	0.00 ± 0.91	0.13 ± 1.15	0.24 ± 1.35	0.23 ± 0.90	0.14 ± 1.08	*0.748*
Calm_T01	3.40 ± 1.25	3.74 ± 1.03	3.09 ± 1.18	3.47 ± 0.97	3.42 ± 1.14	*0.141*
Calm_T02	3.40 ± 0.89	3.74 ± 0.96	3.59 ± 1.00	3.67 ± 0.92	3.58 ± 0.94	*0.452*
Calm_Dif	0.00 ± 1.14	0.00 ± 1.21	0.50 ± 1.35	0.20 ± 1.27	0.16 ± 1.25	*0.291*
Anger_T01	2.31 ± 0.95	2.16 ± 1.16	2.41 ± 1.00	2.30 ± 1.02	2.30 ± 1.02	*0.810*
Anger_T02	1.71 ± 0.94	1.45 ± 0.88	1.68 ± 0.89	1.53 ± 0.82	1.61 ± 0.89	*0.580*
Anger_Dif	−0.60 ± 0.77	−0.71 ± 0.94	−0.73 ± 0.90	−0.77 ± 1.17	−0.69 ± 0.93	*0.868*
Sadness_T01	2.40 ± 0.94	2.32 ± 0.92	2.24 ± 0.98	2.13 ± 0.90	2.28 ± 0.93	*0.655*
Sadness_T02	1.95 ± 0.94	1.71 ± 0.76	1.71 ± 0.97	1.77 ± 0.74	1.80 ± 0.89	0.593
Sadness_Dif	−0.45 ± 0.67	−0.61 ± 0.95	−0.53 ± 0.71	−0.36 ± 0.96	−0.48 ± 0.81	*0.672*
Stress_T01	2.71 ± 0.97	2.35 ± 1.11	2.56 ± 1.02	2.47 ± 1.04	2.54 ± 1.03	0.503
Stress_T02	2.31 ± 1.00	1.77 ± 0.76	2.03 ± 0.96	1.93 ± 0.74	2.04 ± 0.89	0.071
Stress_Dif	−0.40 ± 1.19	−0.58 ± 1.09	−0.53 ± 0.86	−0.54 ± 1.20	−0.50 ± 1.09	*0.911*

T01: initial emotional stages; T02: final emotional stages; Dif: T02-T01; *p*: probability.

**Table 5:** Pearson correlations (*r*) for the four basic emotions and perceived stress at the end of the urban experience (T02), and comfort and soundscape variables.

T2	Happiness	Calm	Anger	Sadness	Stress
Environmental comfort	0.38 ***	0.21 **	−0.28 **	−0.20 **	−0.42 ***
Acoustic comfort	0.32 ***	0.25 **	−0.25 **	−0.19 *	−0.36 ***
SSC_pleasant	0.37 ***	0.24 **	−0.21 **	−0.28 **	−0.35 ***
SSC_calm	0.40 ***	0.24 **	---	---	−0.24 **
SSC_fun	0.3 ***	0.23 **	---	---	−0.23 **
SSC_lively	---	---	---	−0.18 *	---
SSC_natural	0.32 ***	0.25 **	---	−0.20 *	−0.19 *

*** *p* < 0.001; ** *p* < 0.01; * *p* < 0.05.

**Table 6:** Effect (standardised Beta) of the urban environmental experience (UEE) on the four basic emotions and perceived stress.

Independent Vatriables	Happiness (T02)	Calm (T02)	Anger (T02)	Sadness (T02)	Stress (T02)
Calm (T01)	0.251				
Anger (T01)	−0.204		0.454	0.204	0.332
Sadness (T01)			0.281	0.473	0.229
Stress (T01)	−0.203	−0.380			0.192
Age			−0.240		
Education					−0.211
dB LAeq,mean		0.164		−0.170	
Environmental comfort	0.277		−0.259		−0.338
Acoustic comfort		0.270			
SSC_fun			0.324		
SSC_lively					0.295
F	18.96	13.94	19.14	31.56	25.11
d.f.	4129	3131	5129	3131	6128
*p*	<0.001	<0.001	<0.001	<0.001	<0.001
R^2^ adjusted	0.351	0.225	0.404	0.406	0.519

T01: scores at the start of the EEU; T02: scores at the end of the EEU; SSC: soundscape.

## Supplementary Materials

It is available online at https://www.mdpi.com/1660-4601/16/7/1284/s1.
