# Supplementary material for: Going beyond Quietness: Determining the Emotionally Restorative Effect of Acoustic Environments in Urban Open Public Spaces
Source: Int J Environ Res Public Health. 2019 Apr 10;16(7):1284. doi: 10.3390/ijerph16071284 (PMC6479382; doi:10.3390/ijerph16071284)
Supplement: Supplementary file 1 [file ijerph-16-01284-s001.pdf]

Q1-2013-Vitoria-Gasteiz	Code survey					

The identifier must be a real email address. Try using a find it EASY TO REMEMBER.

Enter your ID (PERSONAL_ID)

Part 1. Socio-demographic Data of the participant

In this section we ask you some demographic data.

All the information you provide in this and the following sections will be treated with the utmost confidentiality, being the anonymous questionnaire.

Thanks for your participation.

[0.X

**0.01** Age:		**0.02** Sex:	1 □ M	2 □ F	**0.03** height (cm):		**0.04** weight (Kg):	

**0.05** Education (complete studies)	1- primary	2- secondary	3- university degree	4- university degree	5- Other________________
**0.06** Employment Status	1- Active*	2- unemployed*	3- Student	4- Retired*	5- other **0.07** Indicate profession ________________________

][1.2

Residence: **0.08** town: **0.09** Postal codex

0.10 Residence time in Vitoria-Gasteiz

Part 2. Health and Life Styles

Here are some brief questions about how they perceive their health...

[2.Page 1

2.1. In general, how do you describe your health?

very bad	1	2	3	4	5	excellent

2.2. And, your stress level (last month)?

the lowest imaginable	1	2	3	4	5	the greatest imaginable

] [2.Page 2

2.3. Regarding the following emotions, would you say the last month has felt.

the lowest imaginable						the greatest imaginable
happiness	1	2	3	4	5	happiness
anger	1	2	3	4	5	anger
calm	1	2	3	4	5	calm
sadness	1	2	3	4	5	sadness

][2. Page 3

Comments and suggestions

____

____

]

Q1-2013-Vitoria-Gasteiz	Code survey					

Part 2. Experiencia y Percepción Global con el Lugar

Just get to one of the selected places in the European project Citisense to value...

[3.Page 1

**3.1. Places**	A. Los Herran	B. Park Salinillas	C. Plaza Constitución	D. CEA area

3.2. **Point**	

] [3.Page 2

3.3. **RADIATION (I)**	1-□ Sunny 0-□ Shadow

3.4. **RADIATION (II):**	______% % cover of sky (please do not use decimals)

] [3.Page 3

3.5. How long you been here? _____min.

1 ❑ 0 – 15 minutes
2 ❑ 16 – 30 minutes
3 ❑ 31 – 60 minutes
4 ❑ 61 – 120 minutes
5 ❑ more than 120 minutes

3.6. How long have you been "outside" since he left the last time indoors? ____min.

1 ❑ 0 – 15 minutes
2 ❑ 16 – 30 minutes
3 ❑ 31 – 60 minutes
4 ❑ 61 – 120 minutes
5 ❑ more than 120 minutes

] [3.Page 4

3.7. Where do you come?

1-□ home
2-□ Works place
3-□ geographical place (neighbourhood...)
4-□ study place
5-□ public transport (bus, metro...)
6-□ bars, restaurants...
7-□ shopping, markets, banks...
8-□ Park, garden
9-□ other (specify): ______________________________

] [3.Page 5

3.8. **What activity were you doing just before this survey?** [You can mark several options]

1 - □ sitting
2 - □ reading
3 - □ standing
4 - □ walking
5 - □ going from one place to another (walking)
6 - □ running, walking fast
7 - □ conversing with others
9 - □ Other (specify) __________________________

] [3.Page 6

3.9. How would you describe this place? [Using the following pairs of adjectives, where 1 represents the one on the left, 5 which appears to the right, and 3 neither]

9.1	very unpleasant	1	2	3	4	5	very pleasant
9.2	very dirty	1	2	3	4	5	very clean
9.3	inaccessible	1	2	3	4	5	accessible
9.4	very unsafe	1	2	3	4	5	sure
9.5	very noisy	1	2	3	4	5	very quiet
9.6	very dark	1	2	3	4	5	bright
9.7	very cold	1	2	3	4	5	very warm
9.8	ugly	1	2	3	4	5	very nice
9.9	no landmark	1	2	3	4	5	emblematic
9.10	ugly	1	2	3	4	5	very nice
9.11	boring	1	2	3	4	5	funny
9.12	very artificial	1	2	3	4	5	very natural

][3. Page 7

3.10. Do you use or would use this place to relax, de-stress...?

1-□ Yes
2-□ No

3.11. We could indicate why yes

____

3.12. We could indicate why not

____

][3.Page 8-12

3.13. Could you indicate what your comfort degree is with the following aspects of this place?

	Very low	low	medium	high	Very high
Acoustic	1	2	3	4	5
Thermal	1	2	3	4	5
lighting	1	2	3	4	5
Visual	1	2	3	4	5
overall	1	2	3	4	5

][3.Page 13

3.14. What she likes most about this place?

____

3.15. And the least?

____

][3.Page 147

3.16. Any comments

____]

Q1–2013–Vitoria–Gasteiz	Code survey					

Part 4. Soundscape:
Now, try to focus on what you're hearing...

][4.Page 1-2

4.1. What sounds would you like to highlight from the soundscape of this place? (please only two) and next, could you indicate to what extent you like or dislike them?

Check list (orientative): Traffic, People, Childen, Voices, Music, Nature, Engines…

Remarkable sounds	Very unpleasant	unplea sant	neutral	pleasant	Very pleasant
	**1**	**2**	**3**	**4**	**5**
	**1**	**2**	**3**	**4**	**5**

][4.Page 3

4.2. Now, could you describe the sound environment from your point of view

[Using the following pairs of adjectives, where

1 shows that on the left ;

5 which appears to the right , and

Neither 3]

unpleasant	1	2	3	4	5	pleasant
noisy	1	2	3	4	5	quiet
stressful	1	2	3	4	5	relaxing
discontinuous	1	2	3	4	5	continuous
unknown	1	2	3	4	5	family
difficult conversation	1	2	3	4	5	facilitates conversation
uninformative	1	2	3	4	5	informative
Confusing, chaotic	1	2	3	4	5	clear
common	1	2	3	4	5	characteristic
monotonous	1	2	3	4	5	vibrant
boring	1	2	3	4	5	funny
artificial	1	2	3	4	5	natural

][4. Page 4

4.3. Please close your eyes and concentrate on what you are hearing now. What does it suggests to you? Do not think so much , tell us the first thing that comes to mind …

____
____
____
____
____

][4. Page 5

4.4. Overall, to what extent do you think the sound you hear here is appropriate to take a whole which is environment?

1 – □ very inappropriate
2 - □ Inappropriate
3 - □ neither appropriate nor inappropriate
4 - appropriate
5 - very appropriate

][4. Page 6

4.5. Any comments

____
____

]

Q1-2013-Vitoria-Gasteiz	Code survey					

Part 5. Perception of climate "this place here and now"

[5.1

This section aims to collect what is your perception of the atmospheric conditions at this time.

[5.Page 1

5.1. Could you tell us how the following microclimate conditions are here for you…

a) *Temperature*

1 - very cold
2 - cold
3 -cool
4 - okay
5 - template
6 - hot
7- very hot

b) *Humidity*

1 - very dry
2 - dry
3 - ok
4 - wet
5 - very wet

c) Wind

1- without wind
2 - breezy
3 - windy
4 – quite windy
5 – very windy

][5.Page 2

5.2. Overall, how stressful do you consider "microclimate conditions" here at this time?

nothing stressful	1	2	3	4	5	very stressful

5.3. How do you feel now about "micro-climate conditions" here at this time?

very uncomfortable	1	2	3	4	5	very comfortable

][5.Page 3

5.4. At this time you'd prefer…

less heat	*1*	*2*	*3*	*4*	*5*	plus heat
less wind	*1*	*2*	*3*	*4*	*5*	plus wind
less humidity	*1*	*2*	*3*	*4*	*5*	plus humidity

][5.Page 4 VClos

5.5. We could indicate what type of dress has been here

Shirt : 1 □ Short Sleeve (0,15), 2 □ Long Sleeve (0,20)
Pants : 3 - □ Short / Skirt (0,15), 4 □ Normal (0,20)
5 □ Corduroy (0,25)
Jacket : 6 - □ Light (0,25) , 7 - Thick (0,35) □
Shoes : 8 - □ Shoes / Boots / Sneakers (0,04); 9 - □ Sandals / Flip Flops (0,02)
Other : 10 Jersey: □ Light (0,20), □ Normal (0,28), 11 □ Not proceed (0,00),
Other : ______________________

5.6. We could tell how much you're sweating at this time

1 – None
2 – Something
3 – A lot

][5.Page 5 UV effects

5.7. How long you think you can stay here without sunscreen (UV protection)?

1 – nothing
2 - a few minutes (< 5 minutes)
3 - up to half an hour (<30 minutes)
4 - up to an hour < 60min)
5 - no need to protect me from the sun here

□][5.Page 6

5.8. Are you using some type of sunscreen?

[You **can** mark several options]

1 – not
2 – sunglasses
3 - hat or cap
4 – sunscreen
5 - other [specify which] ______________

5.9. How long do you need to be outdoors to get some vitamin D?

1 – A few minutes (less than 15)
2 - 15 to 30 minutes
3 - 30-60 minutes
4 - 60-120 minutes
5 - More than two hours

□][5.Page 7

5.10. How much vitamin D do you think currently produce?

1 - In this country we have no problem
2 - We produce more than necessary
3 - We produce the necessary
4 - We produce less than the required

□][5.Page 8

5.11. Any comments

____

____

Part 6. And finally...

[6.Page 1

6.1. Could you tell us what your emotional state is now on a scale of 1 to 5, where 1 represents the lowest degree of that emotion possible and 5 the larger or largest.

the lowest imaginable						the greatest imaginable
happiness	1	2	3	4	5	happiness
anger	1	2	3	4	5	anger
calm	1	2	3	4	5	calm
sadness	1	2	3	4	5	sadness

][6. Page 2

6.2. And, what is now, at this time, your stress level?

the lowest imaginable						the greatest imaginable
stress	1	2	3	4	5	stress

][6.Page 3 comments

6.3. Any comments

____

____

____

____

Thank you very much for your feedback
